# Representation of negative numbers: point estimation tasks using multi-reference sonification mappings

**DOI:** 10.7717/peerj-cs.2275

**Published:** 2024-08-23

**Authors:** Zico Pratama Putra, Deni Setiawan

**Affiliations:** 1 Faculty of Information Technology, Universitas Nusa Mandiri, Depok, Jawa Barat, Indonesia; 2 Research Center for Nuclear Materials and Radioactive Waste Technology (PRTBNLR), National Research and Innovation Agency (BRIN), South Tangerang, Banten, Indonesia; 3 Jasa Marga Learning Institute, Jasa Marga, Jakarta Timur, Jakarta, Indonesia

**Keywords:** Sonification, Point estimation, Auditory graphs, Non-visual interaction, Negative numbers

## Abstract

In this study, we examine different approaches to the presentation of *Y* coordinates in mobile auditory graphs, including the representation of negative numbers. These studies involved both normally sighted and visually impaired users, as there are applications where normally sighted users might employ auditory graphs, such as the unseen monitoring of stocks, or fuel consumption in a car. Multi-reference sonification schemes are investigated as a means of improving the performance of mobile non-visual point estimation tasks. The results demonstrated that both populations are able to carry out point estimation tasks with a good level of performance when presented with auditory graphs using multiple reference tones. Additionally, visually impaired participants performed better on graphs represented in this format than normally sighted participants. This work also implements the component representation approach for negative numbers to represent the mapping by using the same positive mapping reference for the digit and adding a sign before the digit which leads to a better accuracy of the polarity sign. This work contributes to the areas of the design process of mobile auditory devices in human-computer interaction and proposed a methodological framework related to improving auditory graph performance in graph reproduction.

## Introduction

The expanding range of contexts in which we interact with technology is pushing the boundaries of traditional graphical user interfaces (GUIs). While multimodal computing offers numerous benefits, blind and visually impaired (BVI) users face significant barriers that prevent them from fully leveraging graphical data. This study seeks to make graphical information more accessible to BVI users and to propose a comprehensive methodological framework that enhances interactions with multimodal graphs. Portions of this text were previously published as part of a thesis (https://qmro.qmul.ac.uk/xmlui/handle/123456789/72879).

The research utilizes mobile tablet devices, which are widely used in educational settings and provide a suitable form factor for performing tasks on a surface that is accessible yet offers sufficient space for reasonable graphical resolution.

The goal of this study is to examine whether presenting the auditory graphs using multi-reference sonification mappings improves the accuracy and efficiency of non-visual point estimation tasks compare to with tasks using a single point mapping. A number of alternative representations using reference tones are explored. We examine the effect of presenting more or fewer audio cues on the users’ ability to estimate the location of the point.

The addition of contextual information for data sonification has been shown to improve interaction with auditory graphs (Nees & Walker, 2008). The study by Metatla et al. (2016) used multiple-reference sonification mapping as context information to improve the accuracy of point estimation tasks. Three pitch-based sonification mappings are examined in their study: single point, single-reference, and multiple-reference, which were developed to deliver information on how far away the user is from the source and to assess their impact on how users perform point estimation tasks. In this research, the positive point on an axis has been mapped into a sine tone to the *Y*-coordinate. A numerical function was used to represent the point’s position to frequencies ranging from 120 Hz to 5,000 Hz (for positions ranging from −15 to 15).

However, this multi-reference approach become inefficient as compared to the usual pitch-only mapping. By analyzing the point estimation errors of the participants in the central regions of the scale, the study found that there was a limit indicating that the information provided by sonified reference markers lacked accuracy. Further, for the multi-reference and single-reference mappings, such a limit was different. While the single-reference tone dropped in accuracy as the destination location moved further away from the source, the multi-reference tones kept providing useful information in the middle ranges (Metatla et al., 2016). We go on to propose a new approach that addresses two weaknesses of Metatla’s approach: first, that it becomes lengthy in duration; and second, that it becomes increasingly cognitively demanding when numbers far from 0 are involved. For greater scale, more references are needed to facilitate positioning and direction.

We formulated the following hypotheses in relation to this study:

*(H1): Users will show more point estimation errors when using the single point sonification mappings compared with the single reference and the multi-references mappings using the step20 and step10 sonification mappings.*

*(H2): Users will show more point estimation errors when using the single reference sonification mapping as compared with the multi-reference mappings of 20 steps and 10 steps.*

*(H3): Users will show more point estimation errors when using the multi-reference mapping of 20 steps compared to the multi-reference mapping of 10 steps.*

*(H4): Users will show better polarity sign selections when using the single point and single reference sonification mappings compared with the one-reference and the multi-references mappings of 20 steps and 10 steps.*

*(H5): BVI participants will perform better on point estimation tasks compared to sighted participants in all conditions.*

The structure of this study is organized as follows: We begin by reviewing relevant literature to lay the foundation for our experimental study. We then detail the empirical research conditions and present the results. Following this, we analyze and discuss the outcomes in depth. Finally, we conclude with a summary of the findings and an overview of the study’s limitations.

### Theoretical background

#### Auditory graph interface

The study of auditory displays, which deals with the use of non-speech sound to display information, has become very active in the last thirty years. The implementation of auditory displays has been engaged in a variety of complex work environments, ranging from computer applications, aircraft cockpits, medical workstations, and control centres of atomic reactors. The main goal in designing these auditory displays is the optimization of the level of conformity between the intended information and the information obtained by listeners using their cognitive experience.

Sonification is a specific type of auditory display, a process typically used to map data sets to acoustic parameters in order to make the data audible (Walker & Nees, 2011). The audio is used to help users evaluate the trend of the data and its distribution while listening to the sound generated as a representation of the rendered acoustic data. Data presented using sonification has a benefit to be perceived as it is broader and clearer than the speech sound which is precise and demands more focus (Brewster, 2002).

Metatla et al. (2016) explored further support of non-visual point estimation tasks using another form of sonification by integrating multiple tones as references to represent a note. Their findings showed that using multiple references in auditory graphs could improve the accuracy of point estimation tasks. It showed that the display of data in auditory graphs using contextual information is more effective if it is properly designed. More research is necessary to explore potential approaches for context implementation that will allow users not only to perform trend analysis tasks, but also to perform point estimation tasks effectively (Metatla et al., 2016).

The potential of auditory representations is not limited to data accessibility for BVI users but extends to other fields, including astronomy. Zanella et al. (2022) highlighted the growing use of sonification in astronomy to represent complex datasets and enhance scientific discovery. They noted the multidimensionality of sound and the human ability to filter signals from noise as key advantages of sonification. Additionally, they emphasized the importance of sound-based techniques in making astronomy more accessible to blind or low-vision individuals, thus promoting their participation in science and related careers. Zanella et al. (2022) also discussed the creation of engaging multisensory resources for education and public engagement, demonstrating the broader applicability and benefits of auditory data representation.

In a similar vein, Cantrell, Walker & Moseng (2021) introduced the Highcharts Sonification Studio, an online, open-source, extensible, and accessible data sonification tool developed through a collaboration between Highsoft and the Georgia Tech Sonification Lab. This tool leverages advances in auditory display and sonification research, and builds on over 20 years of experience from the Sonification Sandbox project. The Highcharts Sonification Studio emphasizes usability and accessibility through an iterative design and evaluation process, highlighting its potential for growth in research, art, and education.

By incorporating lessons from Metatla et al. (2016), Zanella et al. (2022) and Cantrell, Walker & Moseng (2021), our research aims to push the boundaries of auditory graph interfaces. We strive to make graphical information more accessible and usable for BVI users, while enhancing the overall utility of sonification in scientific research, education, and various other fields.

#### The role of negative numbers for auditory graphs

Most studies to date that examined numbers have concentrated on positive numbers, whereas those working on negative numbers are limited. The question that researchers discussed in negative numbers is if they are rendered mentally to their components values or to their holistic values (Fischer, 2003; Pinhas & Tzelgov, 2012; Shaki & Petrusic, 2005; Tzelgov, Ganor-Stern & Maymon-Schreiber, 2009).

This representation of components suggests that the sign and the numeral of negative numbers are treated separately at first. Later on, the meanings of the negative numbers are assembled. When negative numbers are processed, the digit component is only represented on the mental number series, on which the numbers are displayed in order of size, that means smaller numbers are placed to the left of the larger numbers (Rugani et al., 2015). In this way, it would display the negative numbers with a large numerical value (for example, −1 and −2) to the left of the negative numbers with a small numerical value (for example, −8 and −9), which is the opposite of how the positive numbers are displayed (Zhang, Feng & Zhang, 2019).

The polarity and magnitude information of negative numbers, on the contrary, is combined and displayed in the left continuation of the entire series of mental numbers (Fischer, 2003; Varma & Schwartz, 2011). When processing negative numbers, their meanings are called up rather than put together. In this way, if the negative numbers have a large numerical value (for example, −1 and −2), they would be displayed directly to the right of the negative numbers with a small numerical value (for example, −9 and −10), and this would correspond to positive numbers.

While the aforementioned proposed to display negative numbers with respect to their elements in the visual modality, the study by Kong, Zhao & You (2012) investigated how negative numbers are processed in the auditory modality and how it is influenced by context. In one of their investigations, a stimulus recognition task was used in which negative as well as positive numbers were combined as indicators. In this study, an inverse attention SNARC effect was obtained for negative numbers. Their results indicate that in auditory modality, negative numbers are constructed from the set of positive numbers, which supports a representation of the components concept.

#### Mobile multimodal interfaces for BVI

Navigation on mobile devices has been a subject of interest for both sighted and visually impaired users. Various studies have explored different approaches to facilitate non-visual interaction with mobile devices (Amar et al., 2003; Li, Baudisch & Hinckley, 2008; Sánchez & Maureira, 2007; Zhao et al., 2007). With the Mobile ADVICE system, Amar et al. (2003) combines a scroll wheel with acoustic and tactile feedback to browse through the menus of mobile phones. The EarPod, incorporating a circle-shaped touchpad combined with acoustic feedback to facilitate non-visual interaction with multi-level menus, was investigated by Zhao et al. (2007). They claimed that the system outperformed visual menus in practice. Most of these studies require the designer to customize the hardware that is not widely practiced for users in general. Without modifying the hardware, Sánchez & Maureira (2007) has developed mobile and desktop applications that utilize pointing gestures to make it easier for visually impaired users to travel by light rail.

In Kane, Bigham & Wobbrock (2008), slide rules were developed that enable multi-touch gestures combined with audio output when interacting with mobile devices. Further, Kane et al. (2011) have proposed a new framework to facilitate access to mobile devices using gestures on mobile touch screen devices. The BlindSight system by Li, Baudisch & Hinckley (2008) is based on the mobile phone’s physical keypad and is designed to provide access to a non-visual menu during the phone call.

A preliminary study by Metatla et al. (2014) investigated non-visual menu navigation regarding completion times and mental workload. Nikitenko & Gillis (2014), in his work on sonification on mobile touchscreen devices, suggests that audio playback and user interaction combined procedures offer an advantage over procedures that rely solely on audio. Goncu & Marriott (2015) developed GraCALC, a tool for implementing statistical and numerical graphics on mobile devices for the visually impaired. Their system displays a line graph generated from a mathematical function which can be accessed from a web.

Furthermore, the Apple Swipe game uses bidirectional sounds to enable users to play the game without relying on visual cues. This game was designed to be simple and easy to use, with a focus on minimizing latency and utilizing audio cues (Mohd Norowi, Azman & Wahiza Abdul Wahat, 2021).

This research builds on our previous study, “Graph Reproduction Task on Mobile Auditory Graph (MAG): An Exploratory Study”, which investigated the feasibility of using mobile auditory graphs for BVI users (Putra, Sutarto & Anasanti, 2021). In that study, we explored basic graph reproduction tasks using auditory feedback on mobile devices, laying the groundwork for understanding how BVI users interact with auditory representations of graphical data.

## Materials & Methods

The purpose of the study is to propose a new method to solve the two weaknesses of the Metatla et al. (2016) method. The first weakness is that the multi-reference approach used in Metatla et al.’s study becomes lengthy in duration, making it less efficient for users. The second weakness is that it becomes increasingly cognitively demanding when numbers far from 0 are involved, requiring more references to facilitate positioning and direction. To improve readers’ understanding of our research purpose, we briefly describe these weaknesses and the experiment by Metatla et al. (2016). We designed different experimental scenarios to investigate the influence of sonification for better point estimation by adding reference markers. From our point of view, this issue is critical for the auditory display to fit into the broader Human-Computer Interaction (HCI) design space. Some content in this section is derived from our previously published thesis (https://qmro.qmul.ac.uk/xmlui/handle/123456789/72879).

### Apparatus

**Figure 1:**
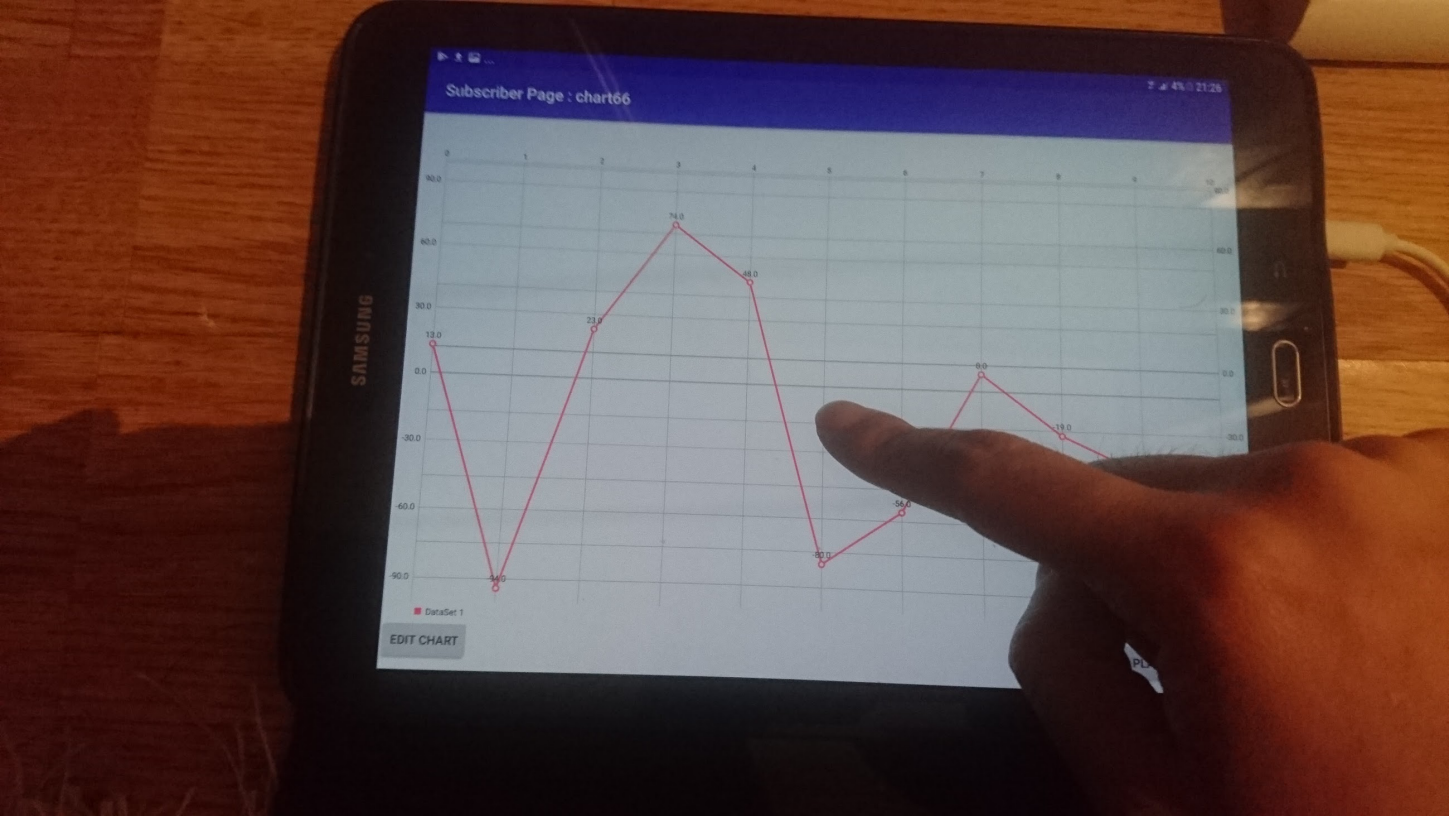
Illustration of the user’s hand interacting on the MAG interface by mobile touch screen gesture.

The Mobile Auditory Graph (MAG) interface was built on a 9.7-inch Samsung Galaxy Tab S2 screen with the Android 7 operating system. A mobile graph application with multimodal input was designed by enabling mobile touch screen gesture interaction for the auditory graph (see Fig. 1).

The prototype has four conditions:

1. Pitch-only, where the point to be estimated is represented by one single note representing its position on the *Y-*axis
2. Single reference, which is similar to condition 1 but each note is preceded by a reminder of the pitch corresponding to *Y* = 0.
3. An approach where notes are played representing each 5th of the distance from 0 to Y-max, up to the value just below the point to be estimated, and then a final note representing the actual point itself. For example, if the notes can range in the scale from 0 to 100, to represent a point to be estimated at 65, points would be played representing the following values: 0, 20, 40, 60, 65.
4. Multiple references with fixed step size, similar to Metatla et al.’s (2016) work. In this condition, notes will be played from 0, in fixed steps sizes of 10, up to the value just below the point on the *Y-*axis to be estimated, and then the note itself. For example, the representation of 65 would include notes for *Y* = 0, 10, 20, 30, 40, 50, 60 and 65. We set Y-max to 100, One single instrument will be used for all displays rather than the two that were used in the previous study.

A pause of 500 ms was used between all the notes representing points in condition 2, 3 and 4, in all sonification conditions. The purpose of these studies is to test the effectiveness of the prototype with the help of sonified graphs in two types of multimodal interaction: playback and swipe.

#### Non-linear human hearing

We used a different pitch mapping scheme that differs from our previous prototype that used linear mapping (Putra et al., 2020). According to Zwicker (1938), frequency range and mapping were designed to conform to the human auditory range, with exponential distribution. Therefore, humans can both determine the pitch and timing of a sound signal with greater accuracy than conventional linear analysis allows. This is also a result of most participants’ concerns about the linear mapping applied to our previous prototype.

The pitch can be regarded as a musical note doubled every 12 semitones which is equal to one octave. A musical frequency assignment can be written as:

(1) \begin{eqnarray*}f={2}^{ \frac{N}{12} }x220\mathrm{Hz}\end{eqnarray*}

where *N* equals the semitone difference of A3 (220 Hz). For each octave, frequency will be doubled (Schiffman, 1977). Table 1 shows the mapping reference ranging from 0 to 100 following the keys of a modern 88-key piano frequency standard, ranging from the 28th key, the C3, tuned to 130.8128 Hz for 0 note to the 79th key, the D ♯7/E ♭7, tuned to 2489.016 Hz for 100. The range and mapping were chosen to fit not only the human hearing range but also to the extent that quasi-melodic processing is engaged by listener stimuli which is a wider pitch range compared to our previous prototype implementing linear mapping.

**Table 1:** Mapping reference value of Y-note with a respective key number, piano key, and its frequency in Hertz. Reference values for Y-notes mapped to corresponding key numbers, piano keys, and their frequencies in Hertz.

**Y Note**	**Key number**	**Piano key**	**Frequency (Hz)**
0	28	C3	130.8128
10	34	F♯3/G♭3	184.9972
20	40	C4	261.6256
30	43	D♯4/E♭4	311.127
40	49	A4 A440	440
50	55	D♯5/E♭5	622.254
60	61	A5	880
70	64	C6 Soprano C	1046.502
80	70	F♯6/G♭6	1479.978
90	76	C7 Double high C	2093.005
100	79	D♯7/E♭7	2489.016

We provided a brief introduction to the use of log/exponential mapping to enhance the accuracy of point estimates. This approach minimizes the usability problem identified in the previous study, as it aligns more closely with the human auditory perception of pitch changes.

#### Representation of negative numbers

Our study proposes a representation of negative numbers in the auditory diagram and uses root mean square error (RMSE) to verify the performance. Since Metatla et al. (2016) did not give a description of the result of polarity data, it is impossible to compare directly. However, one of the main purposes of our article is to propose a novel representation for negative numbers, and we consider finding other experiments for comparison or briefly describing the degree of optimization provided by our method. Negative numbers may be represented *componentially* as two separate components (one digit and one sign) or *holistically*. For this prototype, we chose the *componential representation* approach to represent the mapping by having the same positive mapping reference for the digit and adding one sign before the digit with a “sonar” sound. The sonar sound was chosen because it brings to mind a submarine positioned below 0 m on the land, suggesting the perception of a value less than 0.

#### Touch gesture modality

Touch-based interaction *via* swipe gestures is utilized, allowing users to actively engage with the data by manually navigating through tones along the *Y-*axis. For blind and visually impaired (BVI) users, the interface requires parallel scrolling with two fingers when the screen reader feature is activated, as depicted in Fig. 1. This design allows users to explore auditory graphs through direct interaction, enhancing the comprehension and usability of graphical data for visually impaired users.

### Experimental design

#### Location

This study was conducted in the UK, Germany, and Indonesia, with the entire BVI participants recruited in Indonesia, as it was easier to recruit them there, and they have a solid blind community. We conducted each test ourselves and ensured that the tests were carried out in a quiet room, free from disturbing noises.

#### Preparation and training

After completing a demographic questionnaire, participants were introduced to the concept of sonification, both verbally and using an example, and told that they would be asked to estimate the *Y* coordinates of points on a graph using different sonification schemes. Participants were then introduced to the interface of the MAG app and shown how it is used to display and sonify graphs. Participants could listen as many times as they wished to the different sonification conditions until they felt they understood them. The training period lasted roughly ten minutes for each condition.

The first sonification condition for each participant was chosen randomly. For each condition, the participant listened to the range of possible pitches in increments of ten, going from 0 to the Y_Max_, set at 100 in this experiment. The range of negative pitches was played in descending order, from 0 down to the Y_Min_, set at −100. This test implements the component representation approach for negative numbers to represent the mapping by using the same positive mapping reference for the digit and adding a sign before the digit with a “sonar” sound.

These values were presented with the aim of training the participants’ to differentiate between the sound representing values from the lowest to the highest points in the positive and negative ranges, respectively.

Each condition was tested twice. First, with only multiples of ten; and the second time with sets of numbers randomly distributed spanning each range respectively to expose participants to plenty of values that were not multiples of 10. This procedure continued until all four conditions had been completed.

#### Main experimental session

The experiments were conducted using a Samsung Galaxy Tab S2 on a 9.7-inch screen, running the Android 7 OS. The testing task usually lasted from 10 to 20 min per condition. In this setting, participants could listen to the auditory graph that was played by the researcher. The researcher put a finger on the left-hand side of the MAG app interface that displayed the graph, then moved the finger through the points until the end of the graph. When the researcher tapped a certain point, a participant heard a tone representing the Y-value for the current condition and estimated the point before wrote their estimation on a piece of paper or simply spoke it so that the researcher could note it down.

The participants were asked to finish all four conditions (*pitch only*, *single reference*, multi-reference for steps of 20 or *condition step20*, and multi-reference for steps of 10 or *condition step10*) in random order to avoid possible learning effects. Therefore, one could begin from condition 3, but the next participants could begin from condition 4 and without any sequence of all four conditions. There are 40-point estimation trials for each condition, but the same order of trials applied to all conditions one to four. For example, if condition one began with random trial numbers 23, 35, 9, and so forth; then the condition 2, 3, and 4 would have exactly the same number 23, 35, 9, and so forth.

Participants were asked to perform 40-point estimation trials per condition. Points positions were randomly assigned to ensure comprehensive coverage of points distribution along the axis across the 40 trials. Participants were not given any feedback about the real Y-values after each trial.

At the end of all trials, we conducted informal interviews and asked participants to answer several questions regarding the graphs they had just explored. This questionnaire examined the participants’ difficulties when completing the trials, including the benefits and drawbacks of the multi-reference mode according to the users’ perspective. The overall session, including training and trials, lasted between one hour and 90 min per participant, but took mostly nearer 90 min for BVI participants. In total, we spent 60 h in performing the study.

### Statistical analysis

We used the unbiased root-mean-square error (RMSE) to measure the point estimation error. The RMSE was calculated by taking the differences between values (sample or population values) predicted by a model (standardized estimated or forecasted values = *f*) and the values observed (true values = o) as shown in the Barnston’s formula (Barnston, 1992):

(2) \begin{eqnarray*}{\mathrm{RMSE}}_{fo}={ \left[ \sum _{i-1}^{N} \frac{{ \left( {z}_{{f}_{i}}-{z}_{{f}_{0}} \right) }^{2}}{N} \right] }^{1/2}.\end{eqnarray*}

The statistical results provided in the experiment include the mean, standard deviation (SD), median, and interquartile range (IQR) from point estimation tasks of participants. The mean is calculated as the sum of the errors between the true value and the recorded value divided by the number of observations. The standard deviation is a measure of the amount of variation or dispersion of a set of values. The median is the middle value separating the higher half from the lower half of the data sample. The interquartile range (IQR) is the difference between the first quartile (25th percentile) and the third quartile (75th percentile), representing the middle 50% of the data. This explanation clarifies the methodology for readers.

Further statistical analysis was also performed to find whether any significant differences existed across all conditions of the tasks. Additionally, we considered using covariance to further explore correlations between study results. Covariance measures how much two random variables vary together. By analyzing the covariance between the conditions, we can gain insights into the degree to which changes in one variable are associated with changes in another variable.

### Ethical considerations

The research protects the information of BVI participants in accordance with ethical standards. We ensured confidentiality and ethical handling of participant data throughout the study. Participants were informed about the purpose of the research, and their consent was obtained before participation. All data collected were anonymized to protect participants’ privacy.

## Results

The results are presented according to the guiding questions, offering two distinct scenarios for analysis. Each scenario corresponds to different participant groups: Study 1 focuses on blind and visually impaired (BVI) users, while Study 2 involves sighted users. Portions of this text were previously published as part of a thesis (https://qmro.qmul.ac.uk/xmlui/handle/123456789/72879).

### Study 1: BVI participants’ point estimation with representation of negative numbers

The results were then calculated across all subjects by calculating the RMSE between the estimated values and the true values. For further analysis, the results of the mean, standard deviation (SD), median, and interquartile range (IQR) from 20 BVI participants are calculated as shown in Table 2.

**Table 2:** The comparison of mean, standard deviation (SD), median, and interquartile range (IQR) of root mean squared error (RMSE) from point estimation tasks of 20 visually impaired (VI) participants between four conditions.

**Conditions**	**Count**	**Mean (µ)**	**SD**	**Median ($\tilde {x}$)**	**IQR**
1	20	22.96	11.73	18.31	19.25
2	20	21.51	10.15	20.80	14.18
3	20	17.90	17.41	15.02	7.55
4	20	11.66	8.18	10.24	10.81

These calculations aimed to determine if there are relationships between the performance of point estimation tasks and the condition used to perform the tasks. After calculating the RMSEs, the values were plotted in four boxplots and mean plots to visualise the distribution of the error for each method and each type of graph.

**Figure 2:**
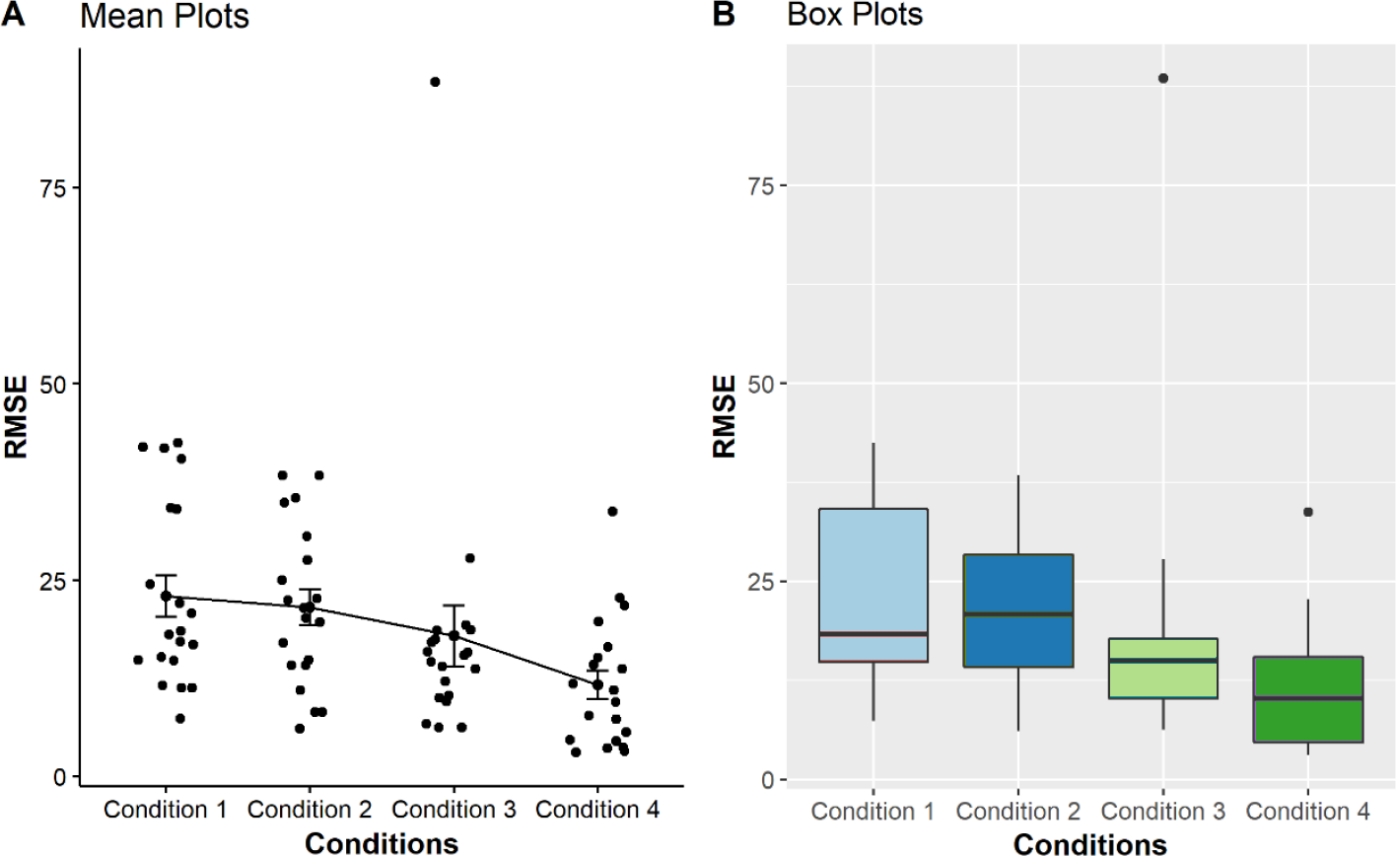
Comparison of four mean plots (A) and boxplots (B) for BVI participants, representing the distributions of RMSE obtained from condition 1 to 4. Condition 1 to 4, *i.e.*, pitch only, zero + single reference, multi-reference for condition step20, multi-reference for condition step10, respectively as displayed on the *X*-axis. The *Y-*axis shows the Mean RMSE values between each error from 0 to 100.

As seen from the boxplots from Fig. 2, the multi-reference mode used in conditions 3 and 4 shows better performances in the point estimation tasks, indicated by smaller distributions as compared with those using pitch-only mode and zero with single reference modalities. We performed a non-parametric significance test with a confidence level *α* = 0.05 to validate our hypothesis for all conditions. *Our null hypothesis is that the RMSE mean of all the conditions are equal*. A Kruskal & Wallis (1952) test was used to check the RMSE mean between the conditions. The test does not assume normality in the data and is much less sensitive to outliers. The calculated *P*-value less than 0.05 led to the conclusion that there are significant differences between the conditions (*p* = 1.09 × 10^−3^).

*Post-hoc* analysis with the Wilcoxon test was performed to determine which levels of the independent variable differ from each other. Adjustments to the *P*-values were calculated to control the familywise error rate (FER) or to control the false discovery rate (FDR).

The pairwise comparison in Table 3 shows that only the pairs conditions 1 *vs.* condition 4 (with *P*-value 0.0061) and condition 2 *vs* condition 4 (with *P*-value 0.0064) are significantly different (*p* < 0.05).

To analyse the performance of BVI participants on sign polarity estimates, their respective values across all BVI participants for all conditions were calculated. We employed a stimuli detection task containing a mix between positive and negative numbers over the 40 trials. The set of positive and negative scores was divided equally between each of the 20 trials in random positions for all four conditions.

We calculated the percentage of correct polarity sign estimates. For example, if all 20 participants correctly predicted negative polarities during a trial of negative numbers, then the percentage is 100. However, if two participants falsely estimated the number as positive, then the percentage dropped to 90. The same rules apply to positive trials.

Separation between four conditions was conducted to determine the relationships of the performance of negative number representation tasks and the condition. Our null hypothesis is that the polarity sign between conditions is equal. The result indicated no significant differences among the conditions (Kruskal–Wallis chi-squared = 7.5951, *p* = 0.052).

### Study 2: Sighted participants’ point estimation with representation of negative number

The results for all subjects were then estimated by evaluating the RMSE between the estimated values and the true values. For further analysis, the results of the mean, SD, median, and interquartile range (IQR) from 20 sighted participants are calculated as shown in Table 4.

**Table 3:** Pairwise Comparisons between Conditions 1, 2, 3, and 4 using the Wilcoxon Rank sum test.

	**Condition1**	**Condition2**	**Condition3**
Condition2	0.797	–	–
Condition3	0.0702	0.0702	–
Condition4	0.0061^*^	0.0064^*^	0.0864

**Notes.**

*p*-value-adjustment shown.

* indicates that a significant difference was found between RMSE means after applying the BH correction.

**Table 4:** The comparison of mean, standard deviation (SD), median, and interquartile range (IQR) of root mean squared error (RMSE) from point estimation tasks of 20 visually impaired (VI) participants between four conditions.

**Conditions**	**Count**	**Mean (µ)**	**SD**	**Median ($\tilde {x}$)**	**IQR**
1	20	30.24	9.86	30.01	10.46
2	20	30.67	7.95	30.28	11.62
3	20	17.44	4.76	16.98	4.90
4	20	12.39	6.31	10.15	9.21

These calculations were made to assess whether there are relationships related to the execution of point estimation tasks and the condition. The results were plotted in four boxplots and mean plots to visualise the error distribution.

**Figure 3:**
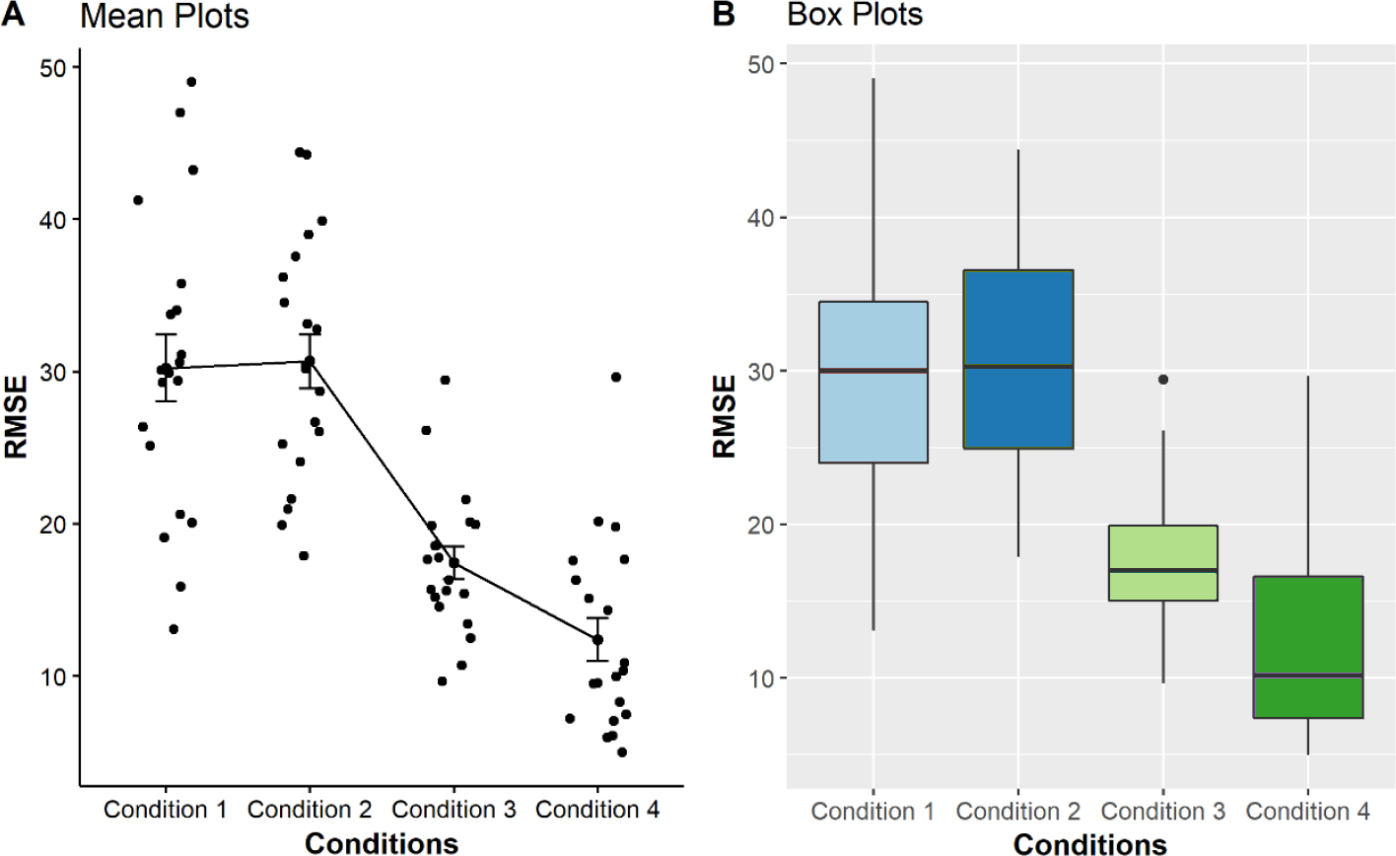
Comparison of four mean plots (A) and boxplots (B) for BVI participants, representing the distributions of RMSE obtained from condition 1 to 4. Condition 1 to 4, (*i.e.* pitch only, zero + single reference, multi-reference for condition step20, multi-reference for condition step10, respectively) is displayed on the *X*-axis. The *Y-*axis shows the Mean RMSE values between each error from 0 to 100.

As seen from the boxplots in Fig. 3, the multi-reference mode used in conditions 3 and 4 shows better performances in the point estimation tasks, indicated by smaller RMSEs are represented with lower median and smaller distributions as compared with those using pitch-only mode and zero with single reference modalities. A Kruskal–Wallis test also identified significant differences among the conditions for sighted users (*p* < 0.001), confirming that multi-reference conditions generally provided more accurate point estimation than other tested methods.

As the Kruskal & Wallis (1952) test is significant, a post-hoc analysis using the Wilcoxon test was performed to determine which levels of the independent variable differ from each other. The pairwise comparison in Table 5 shows that only the pairs condition 1 *vs* condition 2 (with *P*-value 0.82) that are not significantly different (*p* < 0.05).

**Table 5:** The comparison of mean, standard deviation (SD), median, and interquartile range (IQR) of RMSE from point estimation tasks of sighted participants and BVI participants combining all conditions. Pairwise Comparison between Conditions 1, 2, 3, and 4 using the Wilcoxon Rank sum test.

	**Condition1**	**Condition2**	**Condition3**
Condition2	0.82	–	–
Condition3	<0.001^*^	<0.001^*^	–
Condition4	<0.001^*^	<0.001^*^	<0.001^*^

**Notes.**

*p*-value-adjustment shown.

* Indicates a significant difference between RMSE means after applying the BH correction.

The representation of negative numbers was calculated across all sighted participants between conditions to determine whether negative numbers could be represented in terms of their components in the auditory modality using the same scheme with BVI participants.

*Our null hypothesis is that the polarity sign between conditions is equal*. There were no differences between the conditions (Kruskal–Wallis chi-squared = 3.942, *p* = 0.27).

### BVI participants *vs.* sighted participants result

A combined analysis of both groups across all conditions (80 data points) was conducted to determine if there were correlations in performance between the BVI and sighted users (see Table 6).

**Table 6:** Pairwise Comparison between Conditions 1, 2, 3, and 4 using the Wilcoxon Rank sum test. The comparison of mean, standard deviation (SD), median, and interquartile range (IQR) of root mean squared error (RMSE) from point estimation tasks of sighted participants and visually impaired (BVI) participants combining all conditions.

**Conditions**	**Count**	**Mean (µ)**	**SD**	**Median ($\tilde {x}$)**	**IQR**
Sighted	80	22.68	10.86	20.08	14.98
BVI	80	18.51	12.89	15.62	11.36

**Notes.**

*p*-value-adjustment shown.

* Indicates a significant difference between RMSE means after applying the BH correction.

**Figure 4:**
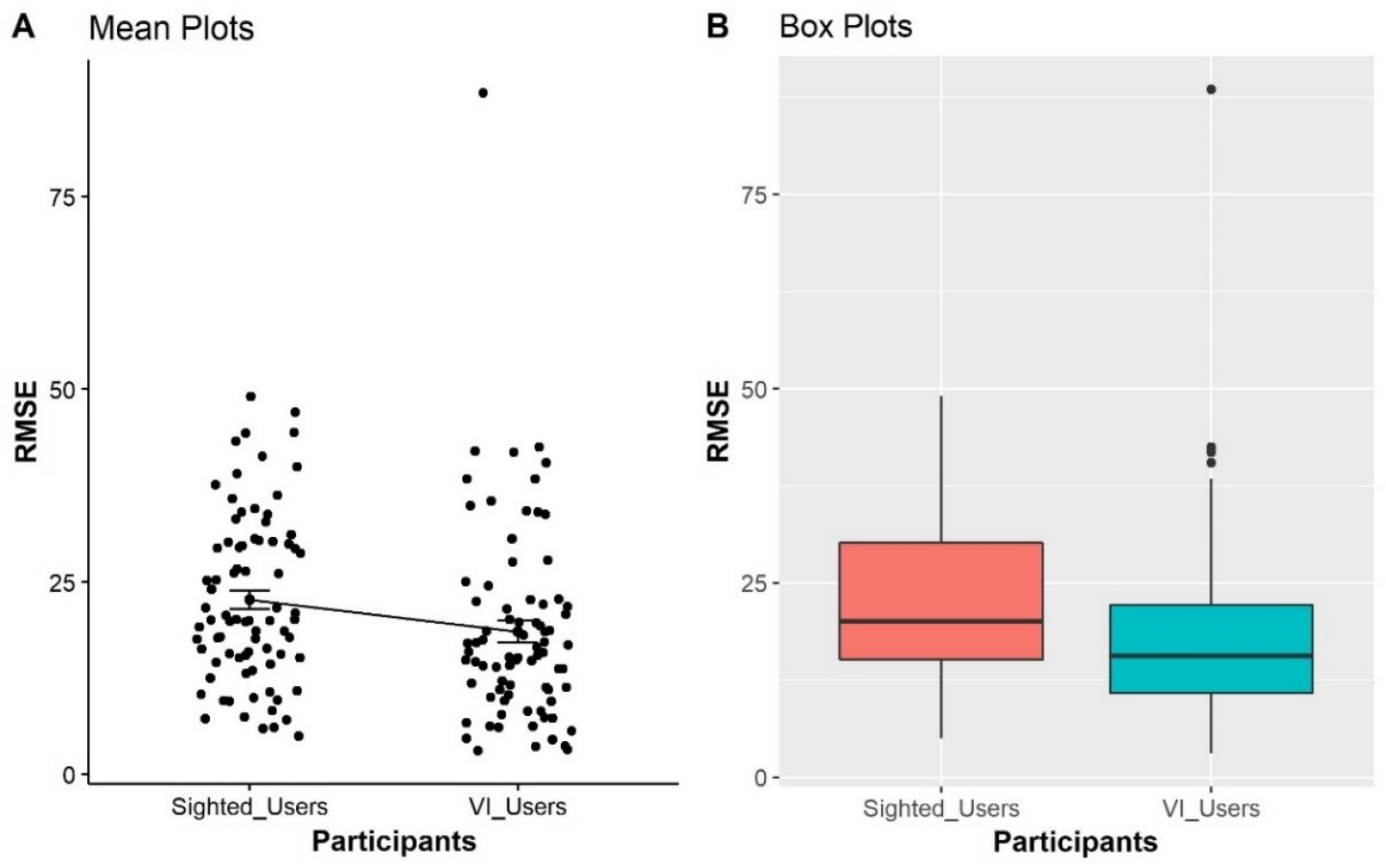
Comparison of sighted participants *vs* BVI participants, representing the distributions of RMSE obtained from all trials from four condition as displayed on the *X*-axis. The *Y*-axis shows the Mean RMSE values between each error from 0 to 100.

As seen from the boxplots in Fig. 4, the BVI participants have better performances in the point estimation tasks in term of lower errors represented with their lower mean and median as compared with sighted participants. It is also interesting that the SD and IQR of the BVI participants were higher, indicating that the point estimation errors are more spread out. As a result, the estimation of the sighted participants has a lower SD, indicating that their error is more clustered around the mean value. A non-parametric significance test with a confidence level of *α* = 0.05 was conducted to validate differences between the users. Two samples of data remain independent if they originate from different populations and the samples do not influence each other. We can use the Mann–Whitney–Wilcoxon test (1947) to determine if the population distributions are identical while not suspecting that they conform to the normal distribution. The differences between sighted and BVI participants in the point estimation task are significant (*W* = 25600, *p* < 0.001).

## Discussion

### Analysis of the point-estimation tasks

Our first research question on the point estimation performance task across four conditions was directed to address our hypotheses *H1, H2,* and *H3*.

In general, the BVI participants produced more accurate results or fewer errors in conditions 3 and 4 which used the multi-reference sonification mappings. This was indicated by its respective lower mean, median and narrower distribution compared with conditions 1 and 2 as shown in Fig. 2. Nevertheless, further *post-hoc* analysis revealed that only condition 1 *vs* condition 4 (*p* = 0.006) and condition 2 *vs* condition 4 (*p* = 0.0064) differed significantly. The other pairs were not found to be significantly different (*p* > 0.05) (see Table 3).

The results for sighted participants showed even greater differences when compared with BVI participants. The accuracy of the multi-reference modes at condition 3 (Median = 16.98) and condition 4 (Median = 10.15) were much smaller, being almost less than or equal half of the RMSE means for condition 1 (Median = 30.01) and condition 2 (Median = 30.28) (see Table 4). Their respective boxplots also showed narrower distributions, as depicted in Fig. 3. This finding was supported by the results of pairwise comparisons which revealed that all pairs were significantly different except between condition 1 *vs* condition 2 (*p* = 0.82) (see Table 5).

Therefore, as predicted in hypotheses one and two, both VI and sighted participants produced higher point estimation errors when using sonification mappings with fewer reference tones (conditions 1 and 2) compared to the multi-reference mappings of 20 steps and 10 steps. These results are in line with Metatla et al.’s (2016) findings who had similar results using narrower scales than the one used in our experiment. It suggested that the multi-reference approaches provide more “anchor” points or more guidance to assist in the point estimation tasks.

Furthermore, *H5* predicted that BVI participants would have better performance on point estimation tasks than the sighted group. As displayed in Fig. 4, the BVI participants performed more accurately on point estimation tasks, represented by a lower mean and median of RMSE values when compared with sighted participants. The result of the Mann–Whitney Test (1947) statistics also showed that the differences were statistically significant (*W* = 25600, *p* < 0.001).

However, when we compared data for each condition separately (see Table 7), it seems that the RMSE values of conditions 3 and 4 for both groups of participants were not different. Meanwhile, in conditions 1 and 2, sighted participants made twice the number of errors than BVI participants. This shows that BVI participants performed more accurately on point estimation tasks with fewer references (*i.e.,* conditions 1 and 2).

**Table 7:** Mean, standard deviation (SD), median, and interquartile range (IQR) of RMSE values on point estimation tasks of VI *vs* sighted participants across all conditions.

**Condition**	**Count**	**Mean (µ)**		**SD**		**Median ($\tilde {x}$)**		**IQR**	
		**VI**	**Sighted**	**VI**	**Sighted**	**VI**	**Sighted**	**VI**	**Sighted**
1	20	22.96	30.24	11.73	9.86	18.31	30.01	19.25	10.46
2	20	21.51	30.67	10.15	7.95	20.80	30.28	14.18	11.62
3	20	17.90	17.44	17.41	4.76	15.02	16.98	7.55	4.90
4	20	11.66	12.39	8.18	6.31	10.24	10.15	10.81	9.21

When the reference was added for every multiple of 20 or 10, both groups showed somewhat equal performance (${\tilde {x}}_{\text{cond.3}}=15.02$, ${\tilde {x}}_{\text{cond.4}}=10.24$ for VI; and ${\tilde {x}}_{\text{cond.3}}=16.98$, ${\tilde {x}}_{\text{cond.4}}=10.15$ for sighted participants, respectively). Therefore, both BVI and sighted groups had similar performance in the multi-reference conditions, with both groups performing rather better in condition 4 (step10 sonification mapping) than condition 3 (step20 sonification mapping). These results tend to show that all participants seemed to perform better as the number of reference points increased, in this case when the range from 0 to Y_Estimate_ was split into 10ths rather than 5ths.

Interestingly, by comparing the SD and IQR of each condition, we observed that the data variability of sighted participants was far lower than that of BVI participants across all conditions.

The participants’ demographic characteristics revealed that several BVI participants a had better level of musical training than the other BVI participants. Our analysis of the data revealed that the more varied data and outliers came from BVI participants with less musical training. Meanwhile, our sighted participants generally had no better musical levels than BVI participants, whose musical level distribution was more varied. Further studies are needed to investigate the possible relationship of *the level of musicianship* for effective point estimation in this context.

We felt the use of the log/exponential mapping made a noticeable difference to the accuracy of point estimates in this study, compared to the accuracy of point estimates in our preliminary studies (Putra, Sutarto & Anasanti, 2021). Although the overall results did not change substantially, this mapping solution effectively addressed usability issues that several participants had previously reported.

We also found no issue in this study as that happens in different timbral streams issue in our preliminary studies. In this issue, participants mistakenly guessed the coin as piano and vice versa, resulting for example, 70 turning to 20 and vice versa. In other words, whether Y_Estimate_ is greater than or equal half of Y_Max_ (Y_Estimate_ ≥ 0.5 * Y_Max_), it could mistakenly perceive as below half of Y_Max_ (Y_Estimate_ < 0.5 * Y_Max_) and vice versa. While it rarely happens in study 3, the solution to use only one type of sound minimizes this potential issue.

### Analysis of negative number reference

Regarding negative number reference, our hypothesis is stated as follow:

H4: & Users will show better polarity sign selections when using the ***single point*** and ***single reference*** sonification mappings as compared with the one-reference and the multi-references mappings of 20 steps and 10 steps.

The results of the polarity sign task revealed no significant difference across all conditions in both in the BVI participants (Kruskal–Wallis chi-squared = 7.5951, *p* = 0.051) and sighted group (Kruskal–Wallis chi-squared = 3.942, *P*-value = 0.267). Table 8 summarizes the descriptive statistics of the percentage of correct polarity signs. Despite the conditions used, both BVI and sighted participants demonstrated high performance in accuracy with all mean values and medians being above 90%.

**Table 8:** Mean, standard deviation (SD), median, and interquartile range (IQR) of RMSE on polarity sign task of VI *vs* sighted participants across all conditions.

**Condition**	**Trial**	**Mean (µ)**		**SD**		**Median ($\tilde {x}$)**		**IQR**	
		**BVI**	**Sighted**	**BVI**	**Sighted**	**BVI**	**Sighted**	**BVI**	**Sighted**
1	40	95.87	95.75	5.76	4.17	100	95	5	5
2	40	94.75	94.13	7.33	5.17	95	95	5	10
3	40	93.62	95.38	9.47	6.03	95	97.5	10	10
4	40	92.12	94.25	7.67	5.13	95	95	6.25	6.25

Metatla et al. (2016), used a *polarity-based approach* using an exponential function—instead of linear mapping—from the smallest negative number to the largest positive number. In our research, however, we attempted to validate that negative numbers are represented mentally in the form of component representation values, not a holistic representation. This is our main reason to implement the *component representation* approach for negative numbers by using the same positive mapping reference for the digit—as described in Table 1—and adding a sign before the digit with a “sonar” sound.

Unfortunately: we cannot compare our results with those of Metatla et al. (2016) because he did not describe the results of the polarity data in his paper. However, it is clear in relation to a weakness in Metatla et al. (2016), that the task of point estimation becomes inefficient as the numbers to be represented get further from 0.

## Conclusions

The work has demonstrated that employing multiple tones combined with an audio component as a representation of negative numbers can assist point estimation tasks for gaining a better perception and interpretation in auditory graphs. The results showed that multi-reference approaches work better than pitch-only or single reference sonification approaches. Both BVI and sighted users performed better on point estimation tasks with the two multi-reference conditions (conditions 3 and 4) than with the pitch-only and single point reference sonification (conditions 1 and 2). The difference in performance was more marked for sighted participants than for BVI participants. We consider that this results from the fact that the multi-reference sonification approaches provide more anchor points (or more guidance) than the pitch-only and single reference conditions.

Further, BVI participants generally perform better on point estimation tasks across all sonification approaches reported here and are more adaptable to different sonification approaches, represented by a lower mean and median of RMSE values compared to sighted participants. However, when we compared data for each condition separately (see Table 7), it seems that the RMSE values of conditions 3 and 4 for both groups of participants were not different. Meanwhile, in conditions 1 and 2, sighted participants made twice the number of errors as BVI participants. This shows that BVI participants performed more accurately on point estimation tasks with fewer references (*i.e.,* conditions 1 and 2).

Both BVI and sighted groups had similar performance in the multi-reference conditions, with both groups performing rather better in condition 4 (step10 sonification mapping) than in condition 3 (step20 sonification mapping). These results tend to show that all participants seemed to perform better as the number of reference points increased, in this case, when the range from 0 to Y_Estimate_ was split into 10ths rather than 5ths.

Comparing the SD and IQR values for each condition, we see that the variability of point estimations by sighted participants was far lower than that of BVI participants across all conditions. The reason why the SD of BVI participants was bigger than sighted participants is due to the fact that we have several long outliers for the BVI participant results. The existence of outliers implicitly shows the diversity of the abilities of the BVI participants.

The component-based approach to representing negative numbers shows a success rate of around 90% in polarity estimates performed by both BVI and sighted users. However, for both sighted and BVI participants, there was some decline in accuracy from condition 1 to 4. We speculate that this might be due to an inverse recency effect, where for those sonification conditions involving more notes (the multi-reference conditions), there was a higher number of instances of incorrect polarity estimates. There was no statistically significant difference between the results achieved by sighted and BVI participants on the polarity estimation tasks.

The studies described here could be extended, for example, to apply to different types of plots other than line graphs such as pie charts and bar charts. It is likely that entirely new or substantially revised sonification approaches than those reported here may be required to render these effectively. Different sonification approaches might be explored for different types of relatively straightforward 2D plots, for example, the efficacy of granular synthesis in representing scatter plots.

A further extension would be to examine approaches to sonifying more complex graphs such as stacked bar graphs and 3D presentation such as 3D spectral plots. Similarly, the research on active interaction methods through multi-touch gestures could be further advanced. As hardware continues to develop, future studies could explore techniques that facilitate the easy comparison of individual point values or comparison of ranges of values using two-handed gestures.

In summary, the above results contribute to the study on non-visual interaction by extending relevant research which is also scalable in the sense that there will never be more than 10th reference tones, no matter what the numbers are on the *Y-*axis with graphs.

## Supplemental Information

10.7717/peerj-cs.2275/supp-1 Supplemental Information 1 Demonstration video of the Mobile Auditory Graph (MAG)

## Additional Information and Declarations

### Data Availability

The following information was supplied regarding data availability: The code is available at Zenodo: - Condition 1: single point mode: zicohasan. (2024). zicohasan/chartMTenLog: Condition 1: single point mode (Study 1, 2, 3, and 4) (DOI). Zenodo. https://doi.org/10.5281/zenodo.11542773. - Condition 2: single reference mode: zicohasan. (2024). zicohasan/chartMTenLogDouble: Condition 2: single reference mode (Study 4) (DOI). Zenodo. https://doi.org/10.5281/zenodo.11542791. - Condition 3: multi-reference mapping of 20 steps: zicohasan. (2024). zicohasan/chartMTenLog30: Condition 3: multi-reference mapping of 20 steps (Study 4) (DOI). Zenodo. https://doi.org/10.5281/zenodo.11542815. - Condition 4: multi-reference mapping of 10 steps: zicohasan. (2024). zicohasan/chartMTenLog01: Condition 4: multi-reference mapping of 10 steps (Study 4) (DOI). Zenodo. https://doi.org/10.5281/zenodo.11542827.
